# Testing the world order: strategic realism in Russian foreign affairs

**DOI:** 10.1057/s41311-021-00285-5

**Published:** 2021-02-22

**Authors:** Scott G. Feinstein, Ellen B. Pirro

**Affiliations:** Department of Political Science, Iowa State University, 503 Ross Hall, Ames, IA 50011 USA

**Keywords:** Russia, Foreign policy, Realism, Near abroad, Global order, Hegemony

## Abstract

Russian actions since 2007 appear increasingly to constitute a realist approach to international affairs. Russia appears to be behaving as a rising power, attempting to become a regional or global superpower. Essential to this pursuit, a state must gather information strategically. We examine one feature of strategic information gathering, called a test, a state action that asserts power but also garners information as it elicits a global response. Through a test framework, we provide a realist explanation to understanding Russia’s foreign affairs and its pursuit of power in the near abroad, Western Europe, Middle East, Asia, and foreign elections.

## Introduction

### Why is Russian foreign policy aggressive?

Thucydides’s ancient claim “The powerful do what they can” is offered as the most obvious explanation. Russian actions since 2007 in Georgia, Ukraine, and even the UK and the Middle East, as those of Thucydides’s Athenians, seem to constitute an offensive realist approach to international affairs (see, for example, Charap and Colton 2017; Mearsheimer 2014; Becker et al. 2016). From this perspective, Russia is behaving as a rising power, attempting to become, at minimum, a regional hegemon in route to being a global superpower.

From a realist perspective, in an anarchic world we must assume that all potential competitor states are revisionists and want to change the status quo (Mearsheimer 2001). In other words, we assume that Russia, as a rising power, is seeking to upset the hierarchy that has the United States as the world’s only superpower and to secure that superpower status for itself. This assumption reflects many of Russia’s expressed contentions. Current Russian president Vladimir Putin has long spoken of dreams of Russian glory. To him, Russia should reclaim its major global position as an equal to the United States. President Putin has expressed his foreign policy goals as centered on the quest for global stability. To realize these ends, he believes a multipolar world is necessary, and Russia must counter U.S. dominance (Chebankova 2017). While many offensive realists explain Putin’s actions in purely power-seeking terms, many of his actions seem unexplained by realist theory. For example, why poison former spies in the UK? Why invade Eastern Ukraine and Crimea and not all of Ukraine?

To explain Russian aggressive foreign policy through a realist lens, we discuss Russia’s actions as a series of tests, illustrating what Russia has learned and suggesting what it may test next. In an offensive realist world, full of uncertainty and dynamic weapons, much remains ambiguous, and the means to the end remain important in understanding realist behavior (Cozette 2004). In this article, we utilize a realist perspective to assess one method of obtaining power—the information gathered in the pursuit of power. While there are many information gathering tools available to states, such as espionage or hacking security technologies like the SolarWind breach of US government materials revealed in December 2020, we focus on tests a state may employ to garner information.

Our examination proceeds in four sections. We begin with a discussion of Russia as a realist actor. Leveraging recent scholarship on Russian foreign policy, we discuss how NATO and EU expansion shaped a realist Russia. Next, we outline our theoretical ideas about realist’s tests, arguing that the pursuit of power is partly enabled by strategic information gathering. After this, we provide an analysis of Russian actions and tests since 2007 that helped gather information in their pursuit of global power over the course of the U.S. Presidential administrations of Barrack Obama and Donald Trump. In particular, we analyze Russia’s “tests” in the near abroad, Western Europe, Middle East, Asia, and elections around the world. We conclude with a prediction of what Russia might test next.

## Russia the realist

“In an anarchic world, [Russia and the United States] have little choice but to compete with the other, lest one fall behind and become vulnerable to the other’s predations” (Walt 2018). Several scholars argue that this competition between Russia and the United States has played out since the breakup of the Soviet Union (see: Kamp 1995; Mandelbaum 1999; Pushkov 1997; Charap and Colton 2017; Mearsheimer 2014).

While a realist Russia may not be inevitable, scholars of offensive realism illustrate well how U.S. post-Cold War policy helped maintain U.S.–Russia foreign affairs in zero-sum terms. Despite initial agreements not to expand NATO to the east, and Russian initial desires under Vladimir Putin to join the EU and/or NATO, the EU and NATO have both denied Putin’s requests and moved eastward at a clip of nearly two countries per year until 2010. The concerns about NATO expansion by foreign policy specialists is well documented (McGwire 1998), and scholarly criticism of expansion is well argued (Kamp 1995; Mandelbaum 1999; Pushkov 1997). George Kennan (Friedman 1998), a primary author of the U.S.’s policy of containment which began in 1947 and adviser present at the formation of NATO, called NATO expansion in the 1990s’, “The beginning of a new cold war,” believing that Russia would slowly react “adversely.” More recently, scholars have described the eastward expansion of NATO and U.S. foreign policy as provocations, leading to Russia’s post-2007 realist-like foreign affairs (Charap and Colton 2017; Mearsheimer 2014). Following NATO’s continued eastward expansion, the bombing of Kosovo, and Western economic and political actions in Russia’s Near Aboard, Russia has indeed begun to act adversely. In this scenario Russia seems to be behaving as if the more power it has, the less power others have to oppose it and its interests. Therefore, as Western allied countries make geopolitical gains eastward, it decreases Russia’s power and ability to pursue its interests, at the very least ensuring that Russian behavior constitutes and reflects an offensive realist policy.

The U.S.–Russian rivalry continues to emerge in several arenas (Samson 2007). For example, there is significant divergence concerning the fate of Syria’s President Bashar al-Assad, with America and Russia initially taking opposing positions. The two states also took antithetical positions toward former U.S. government contractor Edward Snowden, who leaked highly classified information from the National Security Agency in 2013. Snowden fled Hong Kong to Russia, and Moscow granted him indefinite asylum. In response, Washington imposed sanctions on Russia. Most prominently, however, is that the two states cannot reconcile Russia’s annexation of Crimea and its aggression in eastern Ukraine, which triggered additional U.S. sanctions. Toward the end of the Obama administration’s first term, these events reflected worsening U.S.–Russian relations and a continued zero-sum approach to international affairs.

While the recent U.S. retreat from global leadership and abdication of power in several spheres under the Trump policy appears at odds with a realist U.S., they likely add uncertainty to an increasingly uncertain international political arena (Feinstein and Pirro 2019). Traditional U.S., European and NATO alliances are increasingly tenuous. Populists and nationalists in Europe are usurping much of the political dialogue. And China and Iran are finding odd bedfellows in Europe. This change in global relationships only adds uncertainty to the three decades of zero-sum U.S.–Russian political relations.

Most simply, assuming the global system’s perpetual anarchy, the recent increase in uncertainty, and the failure of the West to integrate Russia into the post-Cold War Western international security architecture forced Russia toward expansionism and the pursuit of hegemony (Zevelev 2016; Mearsheimer 2014), Vladimir Putin has advanced quickly in transforming Russia into the major power the USSR once was—a force to be reckoned with by all major actors. His success in achieving this is fostering perceptions of a tri-polar world led by America, Russia, and China.

## Testing the emerging world order: strategic realist theoretical thinking

The anarchic world comes with high levels of uncertainty that force the realist actor to pursue power and gather information along the way. Actors learn what they are able to get away with and how the international system operates, its place within it, as well as its vulnerabilities.

For Mearsheimer, states behave rationally in an anarchic world, one lacking an ultimate authority to call upon to resolve disputes and decrease uncertainty. From this structural position, he and other offensive realists argue that “states quickly understand that the best way to ensure their survival is to be the most powerful state in the system” (Mearshimer 2001, p. 33).

As Kirshner (2012, p. 61) points out, there is a fatal difference “between *being* a hegemon and *bidding* for hegemony.” While it may be ideal to be the most powerful state in the system, “bidding for hegemony is one of the few and rare paths *to* destruction for a great power. Most great powers are extremely likely to survive; most great powers that bid for hegemony do not” (Kirshner 2012, p. 61). In this vein, Kirshner (2012, p. 61) argues that the state should ask, “If I make a bid for hegemony, will I be more likely to survive?” And not, “If I were the hegemon, will I be more likely to survive?” However, accepting Mearsheimer’s (2001, p. 30) assumptions—that structural anarchy, uncertainty of state intentions, security through power as the primary means for survival, and survival the ultimate goal of a state—the rational question lies between these arguments: “How can I survive a bid for hegemony?” The goal is to become the most powerful *and* survive. As Kirshner points out, wanting to be the most powerful and pursuing it is not enough. However, due to uncertainty, abstaining from pursuing power in order to survive is not an option. Consequently, states will challenge the status quo both to pursue power and gain information about how they may gain more power.

Information gathering in the pursuit of power is a necessary endeavor. As Peter Haas (1992) notes, complex issues are difficult to understand. In order usefully to reimagine and conceptualize cause and effect, states and decision-makers are forced to gain tacit information through actions and observations of reactions (e.g., crises, uncertainty, engagement) (Dolowitz 2009). Through practice a state can then obtain tacit information beyond cold facts about power relationships. For example, without context it is difficult to calculate the impact of cyber and biological weapons, stealth technology, and psychological trauma, let alone national unity and resolve that, like an economy, can quickly translate into focused power or disparate into competing parts. Nevertheless, despite the importance of gathering information about these types of hard power, it is largely overlooked by realist scholars (Haas 1992; Levy 1994; Tang 2010). Most pointedly, Mearshimer (2001) assumes that clear information exists regarding where power rests and where one is involved in a zero-sum game. While information regarding military, economic, and political influence may be relatively easy to evaluate,^1^ other aspects of hard power are often best learned through practice (Adler-Nissen 2012; Guzzini 2000; Aron 1966ab).

Experiencing the way the world responds to actions informs a state of global constraints and opportunities, as well as one’s own power. In particular, an aggressive action allows a state to make gains and learn about what actions are most useful for their aspirations. For instance, NATO’s Article 5 stipulates that an attack on one member of the alliance is an attack on all, but what constitutes an attack remains less certain. To clear up the uncertainty, a revisionist state may decide to test the alliance. For example, undertaking cyberattacks on a NATO member state and then observing the degree to which NATO and the U.S. push back would inform Russia about the boundaries of Article 5. In this example, if war is not provoked, the state may learn or confirm that only certain types of attacks are considered “an attack on all.” Similarly, the revisionist state may also learn whether cyberattacks offer sufficient power returns by assessing retributive costs versus the power gained. Finally, observing responses and power returns in practice the state can “interpretatively infer” one’s relative power and what it depends upon (Adler-Nissen 2012, p. 50). As Raymond Aron (1966a, b) convincingly argues, power has no standard value, making power difficult to understand and calculate out of context (Guzzini 2000, pp. 55–60).^2^ However, through practice the state is able to learn more about its power and that of its opponents. For example, following revisionist aggression concessions of power, like bandwagoning and appeasement, are interpreted by other states as signs of relative weakness (Mearshimer 2001, p. 164).

This logic helps explain Germany’s continued expansion in the 1930s and 1940s. Germany tested sovereignty in Europe by invading several countries, including Austria and Poland, producing power gains and information. Through its invasions Germany clearly gained new land and territory. On the information side, the German state could “interpret” Britain’s response—appeasement—as its “unwilling[ness] to defend the balance of power” and Romania’s response—bandwagoning—as having no capacity to resist (Mearshimer 2001, pp. 163–164). In doing so, Germany gained power, gathered information about expansionist opportunities, and learned more about its position in the zero-sum game.

In an offensive realist world, tests are essential to a revisionist state’s security. State expansion is a necessary but highly dangerous pursuit that necessitates information gathering. Tests allow the state to make the necessary power gains and interpret new information about the world and the state’s place within it.^3^ As such, realist tests constitute both an information gathering tool and a pursuit to power. We explore how a realist Russia has been testing the emerging global order and with what results.

## Russia’s tests—the near abroad: Georgia, Crimea, and Ukraine

### Georgia

In August 2008, Russia massed troops on the border between Georgia and Russia. Russia was becoming highly concerned about Georgia’s moves toward the West and in particular the European Union and NATO (Karagiannis 2013). In addition, Russia had been demanding freedom from Georgia for two rebellious provinces, Abkhazia and South Ossetia, which wanted closer links to Moscow. The threat to Georgia seemed overwhelming. Followed by U.S.–NATO military exercises and NATO accession negotiations in Georgia, Georgia attacked. Russia responded with a massive attack pouring over the border and moving toward the Georgian capital. Widely condemned in the United Nations, by the European Union and by most Western states, Russia accepted a ceasefire after changing the sovereignty of these two provinces where Russian troops remain to this day.

Russia had several objectives for its actions. Its main objective seems to have been putting Georgia into a state of semi chaos, without sovereignty over all its territory, in order to thwart the state’s moves to become closer to the European Union and to NATO. Russia regards these Western institutions expanding to its borders as a threat to Russian sovereignty (Charap and Colton 2017). Russia also wanted access to the potential energy resources and port facilities of Abkhazia and ways to control the “energy corridor” from Central Asia, through the Caucasus to the West (See Pallin and Westerlund 2009; Kornely Kakachia 2011; and Cohen and Hamilton, 2011). The “test” initially received minimal pushback in the form of protests and condemnations from the West, and in the longer term, it has produced some negative outcomes for Russia.

After initial occupation Russia did not proceed to annex the two provinces. As the two small, very poor, territories would likely be a drain on limited Russian resources, Russia has opted for diplomatic recognition of these provinces as “nation-states” (something no one else in the world has done). Additionally, in actions reminiscent of the Sudetenland situation before World War II, Russia granted citizenship to residents of the two territories. Russia maintains a military presence in the area as a standing threat to the Georgian government.

Despite Russia’s military successes, the longer range outcome has been exactly what Russia feared. Georgia has signed an association agreement with the EU and applied for membership in NATO, both of which will happen in the distant future. So it seems that Russia’s test was not totally successful, but it did gain much status and respect for Putin’s Russia militarily and somewhat validated the claim of Great Power status as well as limiting Georgia’s stability and regional power.

### Crimea

In 2013–14, Ukraine erupted in demonstrations and riots protesting the failure of its government to sign an association agreement with the EU. The easternmost, ethnically Russian provinces seized the opportunity to rebel against Kiev with the aid of Moscow. During this period of chaos and skirmishes in eastern Ukraine, Russia engineered a takeover of the Crimean peninsula. Utilizing supposed oppression of ethnic Russians, Crimean leaders requested help from Russia and subsequently voted to unite with Russia. While Russian objectives demonstrated Russia’s Great Power status and furthered upheaval in Ukraine, there was a more urgent concern. Russia’s naval fleet was berthed in Crimea, and Russia could not tolerate any threat to the free movement in and out of its ports in the Black Sea. In this case, Russia did annex the territory immediately making provisions for a new bridge directly to Russian territory and opening roadway traffic to Russia.

Widely condemned in the West, the Crimean seizure brought severe economic sanctions to Russia. Yet no other state was willing to mount a counter attack or fight for a free Crimea. So the Russian test was a successful one with the objective of keeping the fleet in Russian waters secured and gaining territory. Russia tested the foundational respect for the sovereignty of Ukraine and seems to have won (Bebler 2015).

### Ukraine

In 2014, there were widespread demonstrations throughout Ukraine condemning the Russian backed government’s corruption and flawed elections. Mired in poverty and lack of development, many Ukrainians favored association with the economic powerhouse of the European Union. Russia, which had meddled in most Ukrainian elections, feared that an EU state on its border was in the offing, and stirred rebellion in the mostly ethnic Russian provinces in the east, Donbas and Luhansk. Civil war followed with Russia moving troops without insignia (little green men) into the region to help the rebels. Despite more sanctions and condemnation, little progress has been made in resolving the conflict. The Minsk accords with both Russia and the EU at the negotiating table froze the conflict in place and sporadic fighting continues to today. Russia’s most feared outcome has happened with the EU signing an association agreement with Ukraine. But the continued fighting and rebellion in the east leaves the situation unresolved, which favors Russia which does not want to annex the area, but wants to keep Ukraine unstable to prevent the West from further gains and to keep the European Union from Russia’s border.

Russia has succeeded in maintaining Ukraine in an unresolved situation, but at a high price. The sanctions imposed by Western powers have severely stunted economic growth and caused widespread suffering. This “test” has been successful by Putin’s standards, but many Russians are beginning to question the need for foreign military actions and desire to stimulate growth in Russia itself to benefit the Russian people.

## Summary

It may seem unusual for one country to invade another and then fail to absorb the territory it comes to control. In these cases Russia seems to have made strategic calculations. To acquire another country would surely provoke a response and a major conflict could ensue—something Russia wants to avoid at all costs, to pick on smaller, weaker neighbors, in which Russia has long had an interest and where ethnic Russians are present is a better bet. Rather than taking over territory, sponsoring close ties produces a group of pro-Russian allies for future ventures. In this area, Russian tests have seemed successful. In other respects, Russia has had to modify its actions. The Georgian conflict, for example, while notably successful, clearly illustrated to the Russian military, as well as outsiders, the limitations and weaknesses of the Russian conflict machine (See Cohen and Hamilton 2011). This led to increased spending on the military and a new emphasis by Putin on nuclear weaponry. There was also considerable outcry among UN members at the Georgian attacks, a superpower overcoming a small country. In the Ukrainian conflict lessons learned in Georgia were put into effect in a modification of the test. Instead of an all-out attack, Russia relied more on subterfuge, using “little green men”—e.g., Russian soldiers without insignia—and ethnic Russian citizens—to achieve its goals. In Crimea, a “vote” by the parliament and a request by the Crimean “government” gave the takeover a patina of legitimacy. Russia worked hard in these three “tests” to achieve its goals with a minimum of negative results and continues to modify its testing actions in light of tacit information received. In these three cases, Russia has tested the international community’s foundational respect for sovereignty in Eastern Europe with the invasion and continued military presence on Georgian territory; annexation of the Crimea; and the incursions and support of the Donbas region of Ukraine’s rebellion against Kiev. The United States and Europe have responded with sanctions and condemnation as well as NATO buildup in Eastern Europe, but have done little or nothing to roll back Russia. This response illustrates that in the emerging international order, traditional expansionism and violations of sovereignty are possible, but not necessarily desirable. Sovereignty violations come with a high price tag, including harm to the Russian economy, and organized opposition from other countries. Nevertheless, the global pushback and support of Ukraine’s Kiev government helped foster two new frozen conflicts in Europe. Going forward, Russia will likely seek to maintain involvement in Ukraine as the chaos provides legitimacy for geopolitical intervention in the region and keeps NATO and the EU further from Russian borders, but, due to the high costs, further expansionism west is unlikely.

## Russia’s tests—NATO and Great Britain: tests of sovereignty, security and espionage

### NATO

Since Russia regards NATO as a primary threat, it is involved in continuous testing of NATO space and defenses. There are numerous examples of these provocations.

In the air, Russian jets frequently violate EU air space (Tomkins 2016). Even the jet carrying Putin to the Helsinki summit (July 2018) with President Trump did not follow correct procedure and was in violation of international law.

In 2016, Russian war ships transited the English Channel on their way to the Syrian coast (BBC, 2016). There have been frequent reports of submarines throughout the Baltic Sea including the state waters around Sweden. A recent occurrence was spotted by schoolchildren (Mizokami 2018).

Russia has been holding frequent military “exercises” where troops are massed in border regions of the Baltic states, Poland and Ukraine. Most recently, Russia has been conducting a massive set of military exercises along with China and Mongolia in the Pacific. These exercises intimidate NATO members, as well as Pacific allies like Japan and South Korea, and demonstrate the Great Power status of Russia. (BBC 2018).

These incidents are not only blatant tests of state sovereignty, but are also threats to the security of the members of NATO and the EU. Russian tests in these areas have been quite successful for Putin. While these incursions are detected, and NATO planes regularly intercept Russian jets, nothing has been done to cause Russia to cease. Publicity, while negative, is further proof to Russia of its great power status. Turkey shot down a Russian plane, which violated Turkish airspace in 2015. No NATO aircraft have either forced down or shot down Russian planes over Europe. This has emboldened Putin to increase the numbers of such incursions.

For NATO, the results have been quite negative. The fact that Russia seems to be able to violate airspace at will reveals ineffectiveness in NATO. Currently, NATO has its difficulties with the Trump administration questioning its very existence. There are also doubts about whether the United States under Trump would come to the defense of smaller countries like the Baltic states in the event of a Russian invasion, invoking Article 5 of the NATO charter.

### Direct violations of sovereignty

Less successful for Russia has been the test of violating sovereignty and security in Great Britain by the attempted assassination of Sergei Skripal and his daughter by planting a highly toxic nerve agent on their doorknob. The resulting outcry internationally has cost Russia dearly in sanctions. Twenty-seven states expelled diplomats in protest. The perpetrators received extensive publicity in the West and it is unlikely they would be welcome in any country (Lawler 2018).

Russian violations of security in the U.S. and Canada also continue to run into trouble, including an indictment of GRU officers for international hacking and disinformation operations, involving cyberattacks and efforts to affect antidoping agencies (U.S. Department of Justice 2018). Far from being able to violate state boundaries with impunity, Russia has been forced into a defensive domestic media campaign and a passel of lies to try to explain away this test.

The rationale for Putin seems to be that Great Powers can go where they want, do what they want, and no one is safe from their reach. It is apparent from the results that Russia has overstepped in this area, and since the events of 2018, it has pulled back from further known violations.

Despite Russia ebbing overt violations of sovereignty, cyber espionage is likely to continue. In December 2020, SolarWinds announced their security services used by the U.S. government had been hacked and compromised. While many attribute the hack to a Russian state backed operation, as of this writing, much remains undisclosed and unclear. Foremost, while it is widely believed that the hack was backed by the Russian state, this has not been publically confirmed. Furthermore, it is unclear whether the hack was a failed clandestine operation or an operation designed to be discovered. What we do know is that the operation directly obtained U.S. government materials and sensitive information, and depending upon the U.S. and global response, the actors will garner more information about what they can get away with. To our knowledge, the United States government has taken no retaliatory action, signaling that this type of espionage is permitted.

## Russia’s tests—the Middle East: tests of security and leadership

The Middle East represents for Russia both a test and an attempt to reshape world order. Putin certainly has tested how much he could get away with in this region. He has supported one of the world’s most notorious dictators in Bashir el Assad against much of the rest of the world who were calling for his ouster. Assad used chemical weapons on civilians against all the norms of international law and has repeatedly attacked his own citizens in his efforts to remain in power. Putin has been able to succeed in Syria beyond any expectations, largely because neither the United States nor Europe wanted to be heavily involved there.

The Trump administration has given much evidence of pulling the U.S. out of the Middle East. While strongly backing Israel, and maintaining a friendship with Saudi Arabia, Trump condemns Iran and removed the U.S. from the nuclear deal. His major concern in the area is the defeat of terrorism and the recent territorial defeat of ISIS satisfies his objectives. In the now seven year old Syrian civil conflict, Trump has moved away from prior objectives. He no longer demands the ouster of President Assad. His support of the Kurds who are largely responsible for the defeat of ISIS has alienated Turkey, which strains the NATO relationship. In the wake of ISIS’ territorial defeat, Trump has no plans to participate in reconstruction, or to be around in the long range abandoning Kurdish allies and moderate Syrians alike.

Into the vacuum left by the United States comes Russia, which sees the Syrian conflict as providing Putin with many benefits. It places Russia as a power player on the world stage. It gives Russia a non-contiguous client state. Russia now has both air and sea bases in the Mediterranean. Now Russia can demand a seat at the table for any peace talks. Indeed the recent Sochi talks about Syria’s future included Iran and Iraq but not the United States (Began July 30, 2018—four rounds to date).

Russia has developed cordial relations with Iran, opened trade, and provided support. Putin welcomed Netanyahu of Israel and they seem to have agreed on trying to eliminate Iran’s troops from Syria or any place near Israel. Russia is working to erode NATO through a developing relationship with Turkey resulting in Turkish purchases of Russian weaponry (incompatible with NATO) and help for Turkey in the face of U.S. sanctions and the resulting financial crisis.

Russia has tested the waters in the Middle East and is quick to expand its influence—in joint military exercises with Egypt, sales of arms to Iran and condemning the U.S. embassy’s move to Jerusalem and validation of Israel’s sovereignty over the Golan Heights.

All of these moves enhance Russian security by drawing neighboring states like Turkey and Iran closer to Moscow, establishing military options and remaking the Middle East into a Russian sphere of interest, a new player in the new world order. And Putin’s leadership in this situation is unmistakable. His foreign adventures are bearing fruit in making Russia a key international player, while his people at home are suffering from the results of the sanctions. There are internal demands for money to be spent on welfare rather than reconstructing Syria. Yet, Putin appears to have benefited greatly from the Middle Eastern tests and expects to reap significant financial benefits from Syria and other Middle Eastern partners, while thwarting U.S. influence in the world.

## Russia’s tests—North Korea and Afghanistan: Déjà vu

In the aftermath of World War II Russia helped create the North Korean state. After the fall of the Soviet Union, its involvement with the North Korean regime was discontinued. Today a resurgent Russia has positioned itself as a benefactor of the Kim dynasty and with a shared border, fosters trade and especially utilizes cheap Korean labor for Russian Far Eastern projects. Russia forgave $10 billion of North Korean debt dating from the Soviet era and has donated large amounts of food during several famine crises.

Nevertheless, Russia does not want nuclear weapons in a state on its border, however friendly it is. It does want to be involved in determining the future of the Korean peninsula. North Korea is less a test for Russia and more a security and push for leadership which will change the future world order in Russia’s favor.

While Russia and the United States agree on the removal of all nuclear weaponry from the Korean peninsula, Russia argues that the U.S. is going about it the wrong way with sanctions and pressure on the North Korean regime. Putin has said that the joint South Korean–United States military exercises should cease. During the last such exercises, Russia flew bombers over the Koreas in an obvious test. To Russia, negotiations should begin immediately with Russia as one of the participants. And the United States should talk directly with North Korea, which would be tantamount to recognition of the legitimacy of the regime. This has happened under the Trump presidency with one on one talks in Singapore (June 12, 2018) and again in Hanoi (February 27, 2019), but without Russian involvement.

The Singapore summit between North Korean dictator Kim Jung Un and U.S. president Donald Trump gave Russia everything it wished for and more. The joint military exercises were canceled. Everyone agreed to denuclearization. And North Korea became more open to the world especially in terms of trade, which Russia will profit by handsomely. Kim subsequently visited Moscow where he was treated like royalty (April 24, 2019). Only recently has the U.S. condemned Russian actions in evading the sanctions to supply North Korea with oil and fuels.

On the whole the Russian state won its test in Korea. It remains to be seen if they will achieve their objectives in remaking world order by bringing the Korean peninsula in line with Russia’s interests.

Afghanistan may be a different story. Russian involvement there was one of the factors precipitating the fall of the Soviet Union. Russia has long maintained its interests there because of its close proximity to Central Asia republics, where Russia has major interests. Putin clearly wants the United States removed from Central Asia, where Russia dominates a number of former Soviet Republics. Afghanistan is a test of whether Russia can resume its involvement in a former client state and remake the situation by ousting the U.S.

Although Trump would like very much to leave Afghanistan, he will only do so with a victory, which does not seem likely given the situation on the ground with the resurgent Taliban and the growing presence of ISIS. Putin’s gamble is to court the Taliban, the very group Russia fought in the 1970′s and 1980′s. They have been given Russian weapons and invited to talks in Moscow. Recent talks had to be called off, however, because the Taliban did not show up. But the Taliban have been talking with the United States (Doha talks 2018–19).

There is no resolution as yet, so we do not know whether Russia’s test has been successful. Working with the Taliban is a bold move on Russia’s part, but the outcome is still undetermined. Moreover, Russia comes up against China’s “Belt and Road” initiative, a global infrastructure project that includes economic and political ties between China and much of Central Asia. Russia is also a part of “Belt and Road,” but has joined for fear of being left out especially because success by China means drawing the Central Asian states including Afghanistan closer to China and further from Russia.

## Russia’s tests—interference in elections around the world

Plagued by democratic deficiencies that justify U.S. and European policy, Russia has used cyber technology to test the legitimacy of liberal democracy and its elections. Through information campaigns, hacking, and financiering, Russia is known to interfere in elections across the globe. Most notably, intelligence agencies agree that Russia actively influenced the 2016 U.S. general election and the UK referendum on exiting the European Union.

In the U.S., election interference proceeded on two tracks. First, intelligence reports documented that Russian hackers had interfered in the U.S. presidential election in several ways. Their objective was to elect Trump and defeat Hillary Clinton. They utilized the Internet and especially social media sites. They hacked the Democrats, planted stories, and spread rumors and false information. They took out ads on Facebook and other social media. There are even indications that attempts were made to hack into voter rolls and voting results in several states. It is hard to weigh the impact these efforts had, but the publicity they received was significant and have led to many questions about the security of the vote and whether or not election results could be trusted.

At the same time, the question emerged whether Donald Trump or any of his associates colluded with the Russians in their efforts to aid his election? Trump denied any Russian involvement in the election calling it “fake news” and misinformation. Putin, too, denied Russian involvement. But the questions persisted, and a series of investigations were begun. Congressional committees have taken up both issues, and a special counsel, Robert Mueller, was appointed by the Justice Department (after Attorney General Jeff Sessions rescued himself) to investigate these matters. Although there were several indictments of Trump associates for wrongdoing, the final Mueller report did exonerate the Trump campaign and candidate of collusion.

In the UK, Russia similarly used multiple methods to interfere in the 2016 BREXIT vote. Russia utilized online platforms and its English language media outlets to push an anti-immigrant and anti-EU narrative. Reports also illustrate that Russian related money funded several political actors.

The electoral interference tests electoral norms in the U.S. and those of its democratic allies, but the most significant effect comes from a two-sided response. First, the Trump administration’s denial of any Russian interference, made it difficult to develop countermeasures to protect U.S. campaigns and elections. One problem is that the collusion investigation overshadowed the problem of Russian hackers, which intelligence analysts claim is continuing in the U.S. and Europe and is likely to continue in upcoming elections around the globe. The Biden administration promises much more security and repercussions if election interference is discovered.

Relatedly, without countermeasures the U.S. Congress has pushed to securitize elections, indirectly questioning their legitimacy. The U.S. Congress and global partners argue that elections have been under attack recently and in need of protection from Russian operatives. The claim is that greater electoral security will ensure the representation of domestic interests.^4^ Nevertheless, securing elections entails some undemocratic behavior, including blaming Russia for American and UK far right nationalism and offering dark espionage and intelligence agencies as the solution to these democratic ills. These moves decrease transparency and the distribution of power, but it is the uncertainty about elections that comes from their securitization that undermines liberal democracy. Asserting that elections are not trustworthy because of Russian interference substantiates claims that at the intersection of information and global perspective rests electoral impropriety. As information and global perspectives cannot be removed from politics, their omnipresence puts into question all electoral outcomes. Consequently, less credible elections shake the foundation of the Wests’ democratic legitimacy, opening opportunities for other regime type claims, including expansionist authoritarianism such as Russia offers. It is impossible at this point to gauge the success of Russian efforts to influence election results, but one can surmise that Russia counts both the Trump 2016 victory in the U.S. and the Brexit situation as “wins.” More successful has been the undermining of the electoral process as a result of all the questioning about Russian interference. Today all election results are suspect and cannot be certified as “hacker” free, helping alternatives—like authoritarianism in Russia—appear less irregular.

## Evaluating Russia’s tests

With the decline of liberal internationalism, Russia is confronting an emerging new world order. Unsure what the new world looks like, how it operates, and what other state responses will be, Russian foreign policy has embarked on a series of tests to determine what is developing. These tests have included conflict, diplomacy, subterfuge, provocations, economic measures, cyberattacks, and utilizing other resources. Most significantly, the tests involved moving troops into Ukraine, seizing Crimea, supporting militarily and financially the leader of Syria, provoking NATO in the air and by sea, getting involved again in North Korea and Afghanistan, and questioning the legitimacy of western elections.

Russia has some clear “wins” in its testing process. Russia achieved its objectives in seizing the Crimea, violating NATO air and sea space, compromising sovereignty in a number of areas, and making great gains in the Middle East. Creating Syria as a client state has been a major victory allowing Russia to claim great power status, acquire naval and air bases on the Mediterranean, and gain a major foothold in the Middle East. It has won because of a lack of response from both the United States and Western Europe. Sanctions have harmed the Russian economy, but failed to cause changes in behavior. In fact, they have emboldened Putin to increase these negative actions.

In other tests, he has achieved only partial victories. He has put into place frozen conflicts in Georgia and Ukraine which have no end in sight. But, in both cases these small countries have responded by moving closer to the EU and to NATO, the very outcomes Putin was trying to avoid. The “blowback” from the Skripal attempt was so negative that Putin seems to be discouraged from further such actions. The verdict is still out in a number of areas. He has partially won in Korea with his announced goals either achieved or at least established as international objectives, although he was not personally a party to any direct actions there. Similarly, in Afghanistan his attempts to insert Russia into the mix have yielded few results, but efforts continue. It is difficult to assess the end results of election interference. This is one area where significant attempts are continuing and expanding. The refusal of the American administration to acknowledge Russian culpability in this area allows Putin to continue his efforts unimpeded and it seems likely that with cyberattacks difficult to combat, there will be greater emphasis in this area.

In themselves, these tests follow realist notions and shape a more zero-sum international order. Not only has a new frozen conflict developed in Europe and Russia gained what Ukraine once had by annexing Crimea, but in the decline of liberal alliances, Russia is making geopolitical moves. Turkey has moved away from the U.S. and closer to Russia by purchasing Russian missile defense weaponry and receiving U.S. sanctions. Similarly, weakened economic U.S.–China economic ties have come, as Russo-Sino military cooperation is on the rise.

Furthermore, this zero-sum approach deepens fractures in Ukraine, propels a security dilemma involving NATO, gains ground in the Middle East, and increases the legitimacy of authoritarian rule. While Russia is likely to end its directional geopolitical march, its gains will likely remain. For the foreseeable future, Russia will hold claim to Crimea and remain a presence in Eastern Ukraine and the rest of the country will remain a political, military, and economic partner with the U.S. and EU. Consolidating gains makes the most sense for Russia. On the one hand, attempts by Russia to gain more regional power verges too close to military conflict. On the other hand, giving up gains jeopardizes Putin’s political standing. For example, Russian concessions in Eastern Ukraine could put Crimea back on the table, an annexation widely popular in Russia and for those who support the Russian president. Relatedly, NATO’s presence in Europe continues to mount, with Trump supporting the Obama administration-led European Reassurance Initiative, a policy that dramatically increased NATO troop levels and spending in Eastern Europe. In reaction, Russia has displayed military sophistication in the region through its 2017 Zapad war games and globally through a strategic submarine navy and 2018 Vostok war games. These military escalations and displays of power in Eastern Europe are likely to continue for the foreseeable future. Russia also has new military bases in the Middle East and Moscow’s influence is expanding, with Assad regaining control of Syria. Finally, as Western elections become less credible, Putin gains new non-democratic opportunities (e.g., either through questionable elections, changing the constitution, informal power grabs, or non-electoral means) to maintain power through 2024: if democracy is illegitimate, why should Russia democratize.

Despite gains, the tests place a strain on Russian domestic politics, which will ultimately determine the power of Russia and the path it pursues. Domestically, Russia’s future international leadership depends upon whether Russians will sacrifice indefinitely or tire of sanctions, and whether the Russian economy can expand beyond the energy sector and become a bigger player in the WTO and global economic fora. The precedent is not good. As oil prices decline and sanctions are levied, there are minimal movements for change and Russian money continues to be invested outside of the country with little expansion beyond energy markets. And despite some domestic shocks, including a rise in the retirement age that led to organized backlash, revolt is contained on two social levels. First, the established generations experienced two horrible shocks with the Soviet Union’s collapse and the economic crippling of the 1990s, supporting a narrative that loyalty is better than revolt. Second, many of the youth coming of age only know Putin and have found a home in his growing cult of personality (Greene and Robertson 2017). While intellectual movements, regional autonomy, and international influence will likely continue to breed discontent in Russia, the rise of far right populism in Europe and questionable elections in the West will continue to undermine small reforms and a potential turn toward revolt or liberal democracy. Furthermore, the Covid-19 pandemic has impressed a unique toll upon Russia that will likely not serve its realist goals. The domestic loss of life and outrage about how the pandemic was handled requires Russian leadership to make moves to regain lost political capital. While gains may be made through geopolitical tactics, pandemic induced economic declines and resources hamper such activities. In consequence, Russia’s domestic conditions do not immediately lend themselves to greater advancement nor retreat in international affairs.

As new government administrations come into office across the world and in the U.S., these test results may vary in how well they hold up. For instance, the Biden administration is much less pro-Putin and arguably more experienced in international affairs than the Trump administration, increasing the likelihood that over the next four years Russia can get away with less. In other words, Russian tests and their results are bounded by global politics.

## Conclusion

To survive in an offensive realist world, a state must gather both power and information in order to be secure.^5^ Mearsheimer (2001, p. 33) maintains that states ensure their survival by being the “most powerful state in the system.” In an extremely unstable and militarized world, only a hegemon knows it will not be conquered. However, while in the pursuit of power and the campaign for hegemony, a state does not want to be destroyed (Kirshner 2012); as such, it has an incentive to accrue power in a manner that does not provoke annihilation. Gathering information about the world—where constraints and opportunities reside, who has power and how much—is essential to becoming a successful becoming a hegemon and not losing in the process (Levy 1994). In other words, “If states are strategic actors, then they must also be learning actors” (Tang 2010, p. 41). While insight might be gained through multiple means, this article demonstrates that strategic actions allow states to gain power without annihilation while accumulating tacit information about one’s power and the international community’s constraints.^6^ The state accumulates power and, in doing so, collects information about what it can get away with.^7^ In the near term, tests will remain an important tool for states. Regardless of ambitions and leadership, a post-pandemic world is uncertain and certainly ripe for testing.

## Footnotes

1 Michael Beckley (2018) provides a compelling discussion of how these aspects of hard power are largely miscalculated.

2 Stefano Guzzini (2000, p. 59) stipulates, “Power comes not (only) out of the utility attached to resources, but exists through the actual value systems of human beings in their relations with each other.” In order to understand power, including how much a state and its opponents hold, necessitates context-based understanding.

3 Similarly, while information gathering through non-aggression is always an option, it does not directly produce power gains or directly reveal clear pathways to power.

4 This is, of course, despite the fact that foreign election interference is a consistent feature in international affairs, as a country’s borders are porous to information and campaign finance.

5 The Covid-19 pandemic likely introduced the greatest amount of uncertainty to global order since World War II. Ignoring borders the virus has brought signs that the post-pandemic world will look both more realist and for others more liberal. For example, the pandemic has brought upon great economic contraction, but what this will entail remains unclear. In the past, economic downturn and loss of life and human capital has led to attempts to concentrate power and great destruction but also perpetuated innovation and cooperation. For instance, the great depression of the 1930s led to global instability and the Second World War, but the great recession of 2008 ushered in eight years of greater cooperation between the US and its allies. Similarly, the Covid-19 pandemic caused a retreat to national borders that constructed greater doubt about the potential for a liberal world order but at the same time facilitated a global cooperative move to contain the spread of the virus. Regardless of a realist or liberal turn, the post-pandemic world is more uncertain and a realist and revisionist Russia will need to undertake more tests to understand its power and place in the emerging world order.

6 While Reiter (1994) notes that large events typically shape the information of states, minimal realist scholarship devotes time to information and strategy (Levy 1994; Tang 2010).

7 This is not to be confused with posturing, an act the state undertakes to *communicate* its power and feelings of threat and uncertainty.
